# Radiotherapy as an alternative treatment option for primary central nervous system lymphoma patients who are noncandidates for chemotherapy

**DOI:** 10.18632/oncotarget.22427

**Published:** 2017-11-10

**Authors:** Yoo-Kang Kwak, Byung-Ock Choi, Kyu Hye Choi, Jong Hoon Lee, Soo Yoon Sung, Yun Hee Lee

**Affiliations:** ^1^ Department of Radiation Oncology, Seoul St. Mary’s Hospital, College of Medicine, The Catholic University of Korea, Seoul, Korea; ^2^ Department of Radiation Oncology, St. Vincent’s Hospital, College of Medicine, The Catholic University of Korea, Seoul, Korea; ^3^ Department of Radiation Oncology, Gyeongsang National University School of Medicine and Gyeongsang National University Hospital, Jinju, Korea

**Keywords:** primary central nervous system lymphoma, lymphoma, radiotherapy, palliation, survival

## Abstract

The standard treatment for primary central nervous system (CNS) lymphoma is based on chemotherapy. However, there are patients who are not indicated for chemotherapy and when left untreated, the expected functional outcomes for these patients are devastating since the disease causes various neurologic symptoms. Therefore, we assessed the effects of radiotherapy as an alternative therapy in primary CNS lymphoma. Thirty-two patients were diagnosed with primary CNS lymphoma and treated with radiotherapy alone. Patients received whole brain radiotherapy (WBRT) to a median dose of 30 Gy (range, 14.4–50 Gy) and the median total radiotherapy dose was 50 Gy (range, 30–54 Gy). The status on neurologic symptoms before and after radiotherapy was inquired during the regular follow-ups. The progression-free survival (PFS) and overall survival (OS) rates for the enrolled patients were calculated. The median follow-up time was 21 months. All but one of the patients presented with neurologic symptoms. The most common symptoms were hemiparesis and headache. After radiotherapy, these symptoms were relieved in 27 patients (84.4%). The median PFS and OS rates were 15.8 and 16.3 months, respectively. Twenty patients (62.5%) experienced recurrent disease at follow up and among them, fifteen patients (46.9%) had intracranial recurrence. The median intracranial PFS was 19.3 months. Untreated primary CNS lymphoma causes neurologic deficits and the survival after only supportive care is poor. Therefore, when chemotherapy is unfeasible, an alternative treatment should be applied and radiotherapy can be a practical option.

## INTRODUCTION

Primary central nervous system (CNS) lymphoma is a rare type of malignancy confined to the brain, spinal cord, meninges, and orbits. Most of primary CNS lymphoma is histologically classified as diffuse large B-cell lymphoma, which has a fast growing and aggressive feature. Likewise, primary CNS lymphoma is characterized by its microscopically diffuse and multifocal feature. In the United States, its incidence has steadily increased since the late 20th century [1]. Yet, it accounts for less than 3% of all primary intracranial malignancies [2]. Because of the rarity of this disease, a standard treatment confirmed by reliable randomized clinical trials has not yet been established. Historically, radiotherapy was the treatment of choice for primary CNS lymphoma [3–5]. However, with the improvement of chemotherapy, the universal treatment used most recently for primary CNS lymphoma is chemotherapy based on a high-dose methotrexate (MTX) with or without radiotherapy. Although studies have demonstrated prolonged progression-free survival in regimens including whole brain radiotherapy [6–9], the major reason for deferring radiotherapy in the treatment of primary CNS lymphoma is the late neurotoxic sequelae induced by multi-modality therapy. Consequently, the actual use of radiotherapy is diminishing and controversy on its role is ongoing. However, there are patients who are reluctant or contraindicated for chemotherapy due to comorbidities, poor performance status, or refusal. The functional outcomes with disease progression for those patients are devastating if they do not receive any treatment. Therefore, a treatment with a palliative aim at minimum must be applied for a better quality of life, and radiotherapy can be a useful treatment option to address this goal. We conducted this study to evaluate the treatment outcomes of primary CNS lymphoma that was treated with radiotherapy in patients who refused, could not tolerate, or were refractory to chemotherapy.

## RESULTS

### Patients

There were 45 primary CNS lymphoma patients who were treated with radiotherapy. Among the total patients, 13 received more than two cycles of chemotherapy and were excluded from the study. Therefore, 32 patients were evaluated. Patient characteristics are provided in Table 1. The median age at diagnosis was 66 years old. Multiple brain lesions were observed in twenty patients (62.5%). The proportion of patients with an ECOG performance status of 2 or 3 was higher (59.4%) than 0 or 1 and poor performance status. The major reason why the patients were non-candidates for chemotherapy was their poor performance status. Nineteen patients (59.4%) were in ECOG performance status of 2 and 3. Five patients (6.6%) were inadequate for chemotherapy due to comorbidities: one patient had poor renal function, one patient had chronic hepatitis B, and three patients had cardiac problem.

**Table 1:** Patient characteristics

Characteristics	Number	%
Age		
< 60 years old	12	37.5
≥ 60 years old	20	62.5
Gender		
Male	16	50.0
Female	16	50.0
ECOG Performance status		
0–1	13	40.6
2–3	19	59.4
LDH level		
Normal	17	53.1
Elevated	15	46.9
Number of brain lesion(s)		
Single	12	37.5
Multiple	20	62.5
Whole-brain radiotherapy dose		
< 30 Gy	14	43.8
≥ 30 Gy	18	56.3
Non-candidate for chemotherapy due to		
Poor performance status	19	59.4
Comorbidity	5	15.6
Progression after chemotherapy^*^	4	12.5
Intolerable to chemotherapy^*^	4	12.5

ECOG: Eastern cooperative oncology group; LDH: lactate dehydrogenase.

^*^these patients received only one or two cycles of chemotherapy due to progression or toxicity.

Twenty patients (62.5%) had multiple brain lesions. Except for one patient, the patients presented with various neurologic symptoms at diagnosis (Table 2). The major clinical presentations were hemiparesis, headache, and memory impairment.

**Table 2:** Neurologic symptoms at presentation

Symptoms	Number	%
Hemiparesis	14	43.8
Headache	8	25.0
Memory impairment	8	25.0
Dizziness	5	15.6
Disorientation	4	12.5
Gait disturbance	3	9.4
Dysarthria	3	9.4
Cognitive dysfunction	2	6.3
Visual disturbance	2	6.3
Seizure	1	3.1

### Treatment

Radiotherapy was delivered at 1.8–2 Gy per fraction, once daily. All of the patients initiated with whole brain radiotherapy (WBRT). Six patients (18.8%) were treated with WBRT only and did not received additional boost RT. Remaining 26 patients (81.2%) received boost RT to the primary mass after WBRT. When planning on the reduced field for boost RT on primary mass, gross tumor volume (GTV) was delineated based on the T1-weighted gadolinium-enhanced image on initial brain magnetic resonance imaging (MRI). Clinical target volume (CTV) was created by expanding GTV with 1–1.5 cm margin in all directions. Another 0.5 cm margin in all directions from CTV was given to create the planning target volume. In patients with multifocal disease, radiotherapy field reduction was performed as follows: when the lesions were distinct and a large distance between the lesions existed, separate fields were used; when the lesions were near enough to the coalesced in the margin, one field was used; in numerous and diffuse lesions, only WBRT was delivered.

The median treatment time was 35.5 days (range 12–48 days). There were no treatment interruptions. Patients received WBRT at a median dose of 30 Gy (range, 14.4–50 Gy) and the total median radiotherapy dose was 50 Gy (range, 30–54 Gy).

### Treatment response

Symptoms caused by the disease were relieved in 27 patients (84.4%). In these patients, symptom relief was observed at median 23 days after radiotherapy started. The response evaluation in all patients was performed with brain MRIs. The first assessment of treatment response was performed at median 4 months (range, 1–9 months). None of the patients presented disease progression at the first assessment after radiotherapy completion (Table 3). Only one patient had a stable disease. Sixteen patients (50%) exhibited a complete response and fifteen patients (46.9%) had a partial response. Most of the patients were tolerable to radiotherapy and reported toxicity is described in Table 4. Of the patients who complained of toxicity, nine patients (28.1%) experienced white matter change in post-treatment brain MRI, but did not require additional steroid therapy. One patient suffered grade 3 general weakness and another patient suffered grade 3 neutropenia. No grade 4 and above toxicity was observed.

**Table 3:** Radiotherapy response

Treatment response	Number	%
Complete response	16	50.0
Partial response	15	46.9
Stable disease	1	3.1
Progressive disease	0	0.0

**Table 4:** Toxicity after radiotherapy according to CTCAE v4.03

	Toxicity Grade			
Non-hematologic toxicity	1	2	3	4–5
Fatigue	2	2	1	
Nausea	3	1		
Headache	1			
Dizziness	1			
Anorexia	1	1		
Total	8 (25%)	4 (12.5%)	1 (3.1%)	0
Hematologic toxicity	1	2	3	4–5
Neutropenia	2	1	1	
Anemia	9			
Thrombocytopenia	1	3		
Total	12 (37.5%)	4 (12.5%)	1 (3.1%)	0

CTCAE: Common Terminology Criteria for Adverse Events.

### Survival

**Figure 1:**
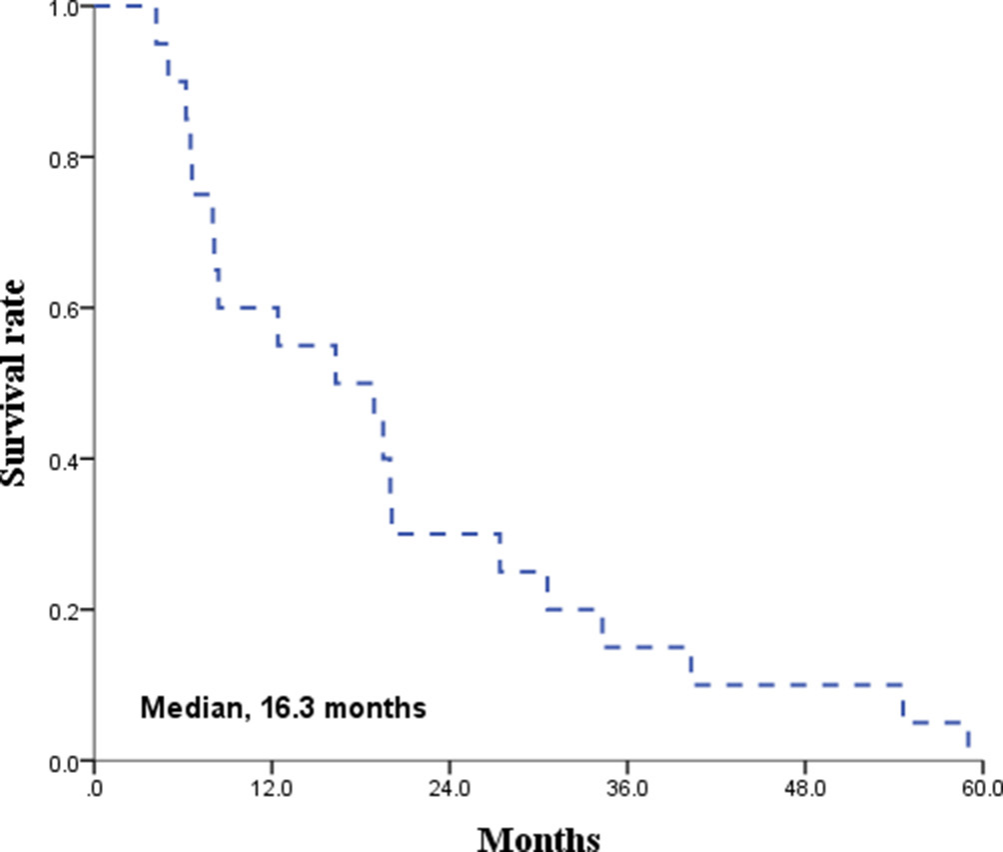
Overall survival rate

**Figure 2:**
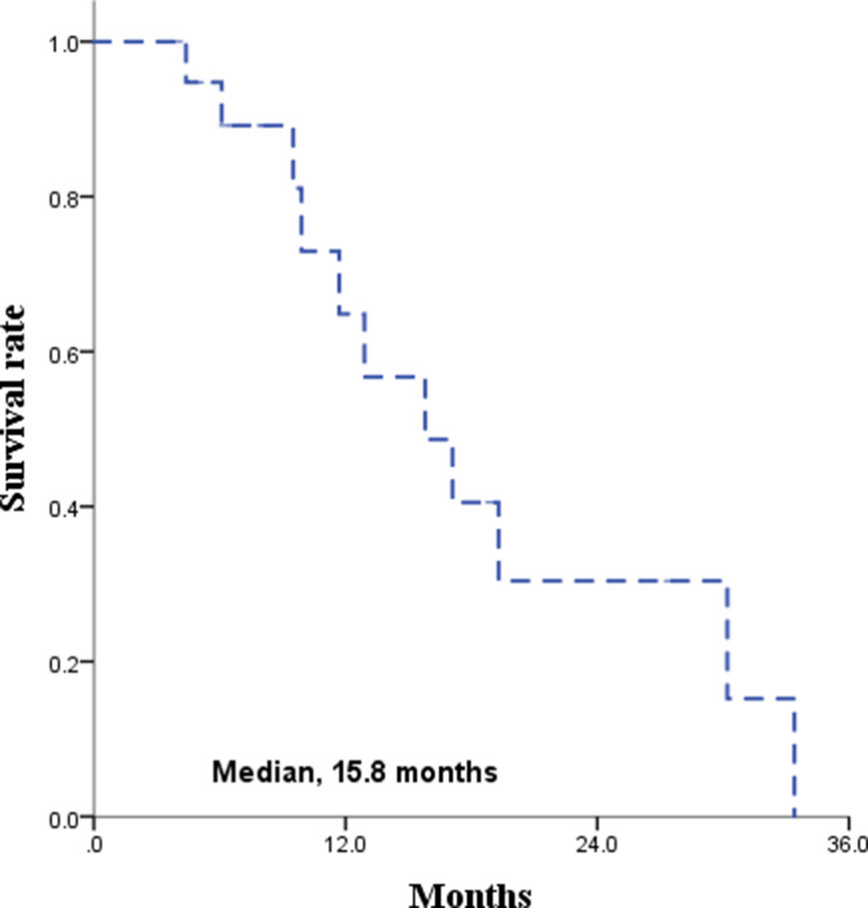
Progression free survival rate

**Figure 3:**
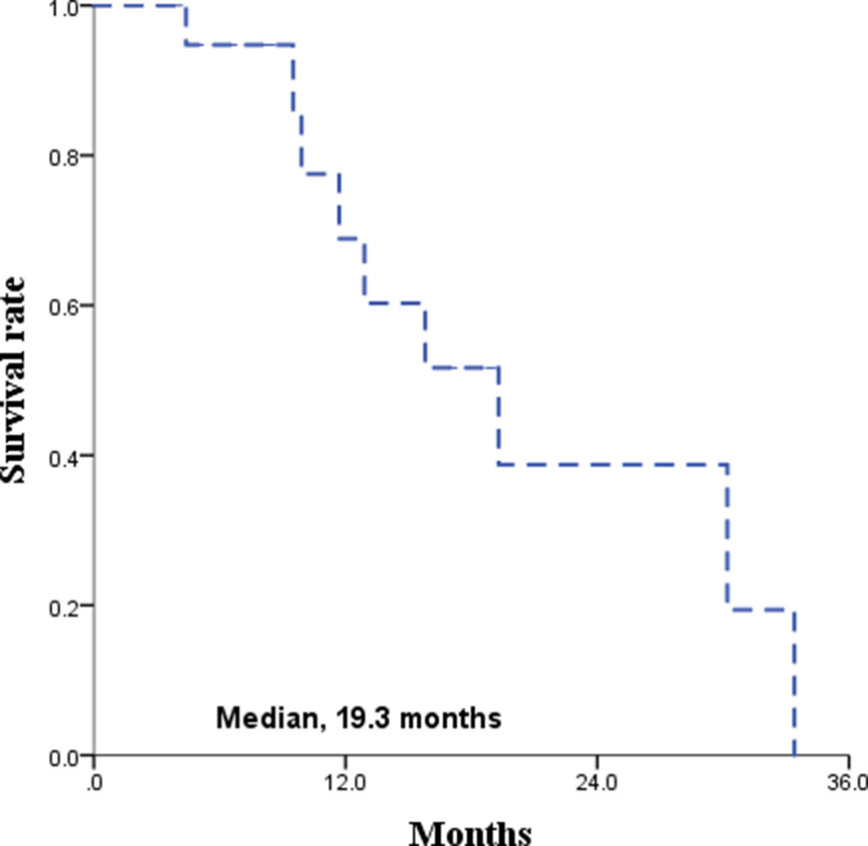
Intracranial progression free survival rate

**Figure 4:**
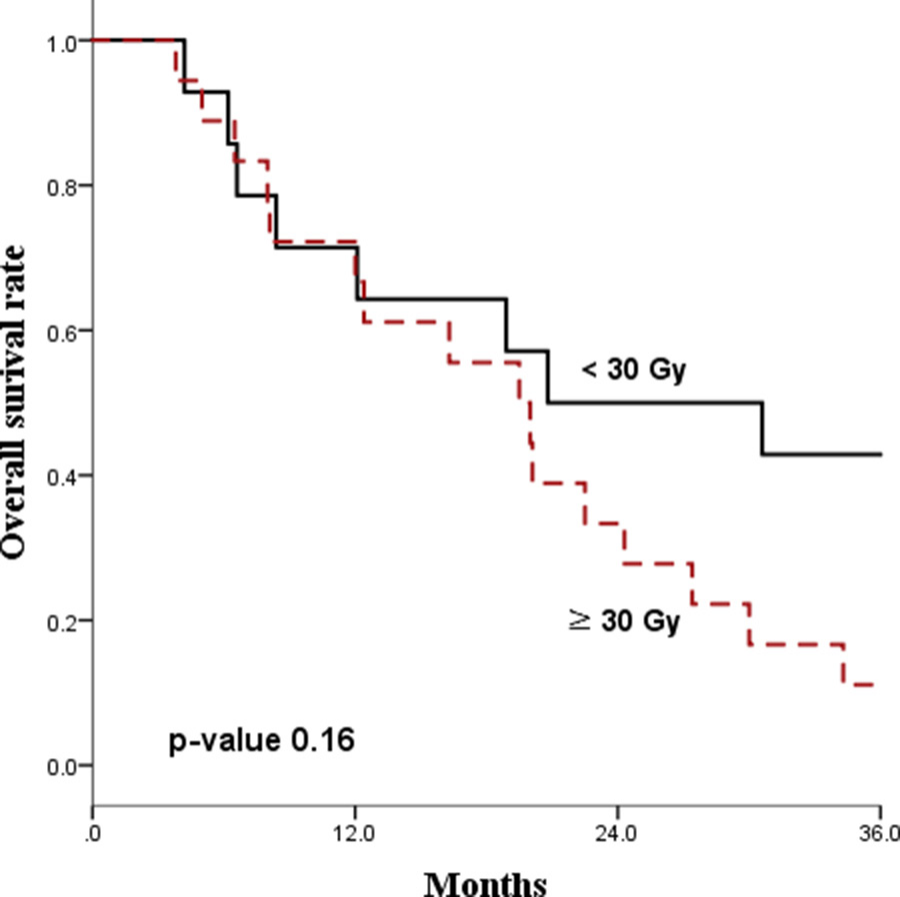
Correlation between whole brain radiation dose and overall survival

The median follow-up time for all patients was 21 months. The Kaplan-Meier survival curves for PFS and OS are displayed in Figures 1 and 2, respectively. The median PFS and OS were 15.8 (range 8.7–22.9) and 16.3 (range, 2.1–30.5) months, respectively. Since radiotherapy is a local treatment modality, we also assessed intracranial progression. Twenty patients (62.5%) showed recurrent disease at follow-up and among them, fifteen patients (46.9%) had intracranial recurrence. The median intracranial PFS was 19.3 (range, 10.2–28.4) months (Figure 3). A correlation between radiotherapy dose to the whole brain and survival was analyzed and the results are depicted in Figure 4. There was no statistical difference between the above and below 30 Gy.

## DISCUSSION

The incidence of primary CNS lymphoma continues to rise [10–12]. The algorithms of primary CNS lymphoma treatments have changed. In the 1970s, primary CNS lymphoma was treated with radiotherapy as a single-modality treatment [13]. Presently, the high-dose MTX based chemotherapy is generally applied in the treatment of primary CNS lymphoma and radiotherapy is often deferred or avoided due to late neurotoxicity. The treatment outcome of patients treated with chemotherapy alone is reported as a median PFS of 21.5 months and median OS of 39.4 months. For patients who achieved a CR after chemotherapy and received radiotherapy, the median PFS and OS was 36.3 months and 38.8 months, respectively [8, 14–18]. Although treating primary CNS lymphoma with chemotherapy alone has a risk of disease progression, the overall survival rate was acceptable and this is the main reason for treating primary CNS lymphoma with chemotherapy.

Unfortunately, there are patients who are unsuitable for chemotherapy due to old age, poor performance status, comorbidities, and/or patient’s unwillingness to engage in chemotherapy. Actually, in a population-base study, the proportion of the patients reluctant to chemotherapy was as high as 39% [19]. For the patients who are inadequate for chemotherapy, secondary treatment options can be radiotherapy, surgery, and conservative treatment. However, most of the patients in this category also cannot tolerate surgery because the patients are in poor general condition in most of these cases. With conservative treatment, including steroid therapy, the median survival is around 3 months due to the disease’s fast growing nature [20]. When the disease progresses, the patient’s expected functional outcome is devastating since the disease involves the CNS; therefore, a secondary or palliative treatment should be applied, and the remaining option is radiotherapy. The treatment duration after WBRT is short due to tumor regrowth and the reported survival with WBRT as a single-modality is 10–12 months [5, 8, 18, 19, 21, 22]. However, the nature of primary CNS lymphoma is not only chemosensitive but also radiosensitive; the tumor response rate after radiotherapy is excellent. Accordingly, neurologic symptom relief occurred in 84.4% and more than 90% of the patients achieved CR or PR in our study. Additionally, the treatment outcome after radiotherapy was fairly acceptable with a median PFS of 15.8 months and median intracranial PFS of 19.3 months.

When a patient is noncandidate for chemotherapy, radiotherapy has more benefits compared to supportive therapy by not only preserving activities of daily living, but also prolonging life. When treated with radiotherapy, a balance between the benefits and risks should be considered and the high incidence of neurotoxicity is the major reason for radiotherapy in primary CNS lymphoma is often deferred or avoided. In previously reported studies [8, 23], WBRT was delivered to a total dose of 45 Gy, which is a relatively high dose and very likely to cause critical neurotoxicity. In 2013, Morris et al. reported on the advantages of reduced-dose WBRTs [24]. WBRT with 23.4 Gy and 45 Gy were compared and the patients who received the reduced-dose WBRT had better PFS and OS with a decreased neurologic toxicity. Although a thorough and routine assessment on neurotoxicity was not available in our study, the reported toxicity was acceptable and these acceptable results may have come from our radiotherapy scheme with relatively low dose to the whole brain with a median 30 Gy. In our study, the correlation analysis between the radiation dose to the whole brain and survival demonstrated no statistically significant difference between the administration of above and below 30 Gy. Since WBRT of 30 Gy and below does not produce fatal neurotoxicity, it can be delivered safely without harming the treatment outcome. To validate the radiotherapy dose and scheme, further investigations with randomized trials should be executed.

Due to this study’s retrospective nature, selective bias and observer bias were inevitable and these yield a limitation to our study. A relatively small number of patients is another limitation and our results should be interpreted with caution. Assessment on patients’ neurocognitive function was not thoroughly performed and the data on toxicity was based on patient-reported symptoms and physician-detected signs such as radiographic changes and the use of medication. Yet, non-hematologic and hematologic toxicity were quite tolerable in most of the patients. Also, since there was no grading system for the severity of neurologic symptoms, the assessment of symptom relief could not be conducted systematically. However, when the neurologic symptoms were relieved after radiotherapy, it was specified in the medical records. This result for which there was an alleviation of neurologic symptoms and acceptable tumor response, radiotherapy in primary CNS lymphoma was worth the treatment when chemotherapy is not indicated.

Since the brain controls most of the activities of the body, the expected outcomes of untreated primary CNS lymphoma are devastating. When chemotherapy is unfeasible, treatment with a palliative aim at minimum, should be administered and radiotherapy is a practical option for satisfying symptom relief and modest disease control. Since the adequate dose for radiotherapy has not yet been established, a study with a large number of patients and long-term follow-up is necessary to determine the optimal radiotherapy scheme.

## MATERIALS AND METHODS

### Patients

This study included data of patients with histologically confirmed primary CNS lymphoma and treated with radiotherapy from January 2005 to December 2015. Inclusion criteria were patients who received less than three cycles of chemotherapy before radiotherapy, age of 20 years or older, no evidence of systemic non-Hodgkin lymphoma, and negative human immunodeficiency virus (HIV-1) infection. Patients with a history of other malignancies were excluded. Age, performance status, history taking, physical examination, complete blood counts, and blood chemistry were evaluated. All patients had neuroimaging with brain computed tomography (CT) and MRI with T1-weighted sequences before and after a contrast injection and T2-weighted images. The work-up included a HIV blood test, lumbar puncture, and ophthalmological assessment. This study was approved by the institutional review board.

### Treatment

Fifteen patients (46.9%) could not receive chemotherapy due to poor general condition. Five patients (15.6%) were unsuitable for toxic chemotherapy due to underlying comorbidities. Twelve patients (37.5%) were treated with chemotherapy at first, but the disease progressed after one or two cycles of chemotherapy.

Radiotherapy simulation was done in a supine position and a u-frame mask was used for immobilization. An enhanced brain CT for simulation was obtained in 3mm slices. For more delicate delineation of the targets, brain MRIs were fused to the simulation CT images. Radiation was delivered at a median total dose of 50 Gy (range, 22–54 Gy) and all patients received whole brain radiotherapy upon initiation. The median whole brain radiotherapy dose was 30 Gy (range, 14.2–50 Gy).

### Assessment

Patients were interviewed weekly during the treatment, monthly for three months after the treatment, and every three months thereafter. Neurologic symptoms caused by the disease were queried before radiotherapy and the changes in the symptoms during and after the treatment were closely observed. A brain MRI was used for response evaluation after the completion of radiotherapy. A complete response (CR) was defined as no remaining mass. A partial response (PR) was defined as a decrease of at least 30% or more in size. Stable disease (SD) was defined as less than 30% decrease in size. Progressive disease (PD) was defined as an increased size of the initial mass or development of any new lesions. Overall survival (OS) was defined as the period from the date of diagnosis to death from any cause. Progression-free survival (PFS) was defined as the period from the date of diagnosis to progression. Assessment on toxicity was done based on medical chart records on patient-reported symptoms, physician-detected signs, and radiological changes in follow up images.

### Statistics

The goal of this study was to assess the role of radiotherapy in symptom relief with functional recovery and tumor response to radiation in primary CNS lymphoma when standard chemotherapy cannot be administered. The OS and PFS curves were assessed with Kaplan-Meier analyses. All statistical tests with *p*-values less than 0.05 were considered statistically significant. All statistical analyses were performed using the R software, version 3.1.2 (R Foundation for Statistical Computing, Vienna, Austria; www.r-project.org).

## Abbreviations

CNS: central nervous system
WBRT: whole brain radiotherapy
PFS: progression free survival
OS: overall survival
MTX: methotrexate
CT: computed tomography
MRI: magnetic resonance imaging
CR: complete response
PR: partial response
SD: stable disease
PD: progressive disease
ECOG: Eastern cooperative oncology group
